# Revisiting Post-Laminectomy Kyphosis and Challenges in Its Management: A Case Report

**DOI:** 10.7759/cureus.62359

**Published:** 2024-06-14

**Authors:** Amol Mittal, Rakesh Mishra, Hrishikesh Patel, Abhishek Shetty, Adesh Shrivastava

**Affiliations:** 1 Department of Neurosurgery, All India Institute of Medical Sciences, Bhopal, Bhopal, IND

**Keywords:** deformity, posterior tension band, posterior cervical fusion, laminectomy, cervical kyphosis

## Abstract

The posterior ligamentous complex plays a pivotal role in spinal stability during complex movements, especially at the cervical vertebral level. Its disruption leads to the development of post-laminectomy kyphosis. The present case emphasizes the challenges in managing post-laminectomy kyphosis, restoring spinal alignment, and the importance of the posterior tension band as a spine stabilizer. A 19-year-old male underwent C2-C5 laminectomy for cervical C3 neurofibroma at an outside hospital. The patient remained stable for five months and then developed cervical kyphosis, leading to myelopathy. Clinical examination revealed significant neurological deficits, including spasticity, clonus, loss of hand dexterity, and sensory abnormalities. Imaging revealed C3 retrolisthesis with severe cervical kyphosis, cord compression, and myelomalacia. The management involved cervical traction with gradual increments in the weight and correction of the cervical sagittal balance. Principles of kyphotic deformity correction were applied, and C2 pedicle with C3-C5 lateral mass fixation was performed. The patient's modified Japanese Orthopaedic Association score improved from 10 to 16 at six months’ follow-up. Post-laminectomy, the disruption of the posterior ligamentous complex increases the range of motion, particularly in the cervical spine, leading to instability and kyphosis. Surgical interventions such as laminoplasty, laminotomy, and laminectomy with posterior cervical fusion aim to mitigate the risk of kyphosis, with techniques such as bone-to-bone ligament-preserving laminoplasty and ultrasonic bone scalpel showing promise in further reducing the risk of kyphosis. The key determinant for the prevention of kyphosis is the integrity of the posterior ligamentous complex. The management of cervical kyphosis includes appropriate pre-operative planning, which includes the evaluation of cervical and spinopelvic parameters. For a posterior spinal approach, one may choose to consider laminotomy, laminoplasty, or laminectomy along with posterior cervical fusion.

## Introduction

Adult patients who undergo intradural tumor resection have an 11% to 56% chance of developing iatrogenic spine deformity [1,2]. The incidence is higher in the pediatric age group [3]. The posterior ligamentous complex (PLC) accounts for 30%-40% of spinal stability during rest, and this percentage is largely based on the inherent biomechanical characteristics of each ligament [4]. The role of the PLC is much more crucial in maintaining stability at the cervical segment compared to other spinal segments. Multilevel laminectomy and laminoplasty for excision/decompression in case of spinal malignancies, spinal stenosis, disc herniation, and so on cause PLC disruption, which leads to the development of kyphosis. The PLC is prone to non-healing. In addition, the loss of the posterior cervical muscles and the redistribution of loads via the posterior facets and anterior vertebral body due to the PLC disruption are significant contributors to the development of post-laminectomy kyphosis (PLK) [5].

The present case emphasizes the challenges in managing PLK due to the loss of PLC as a major spine stabilizer following multilevel laminectomy. It signifies and reaffirms the importance of maintaining the integrity of PLC and opting for laminoplasty or fixation post-laminectomy to minimize complications such as PLK.

## Case presentation

**Figure 1:**
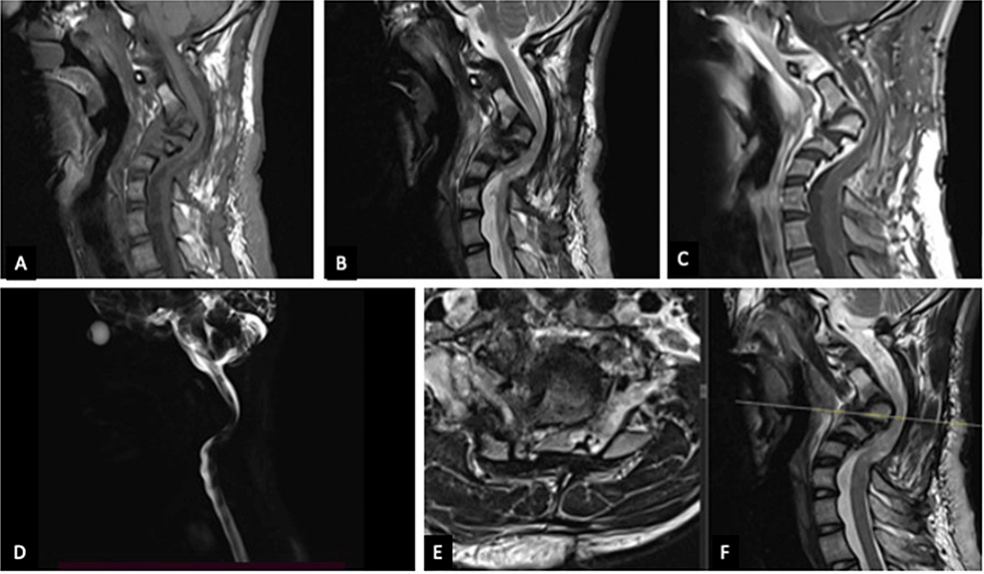
MRI of the cervical spine showing severe retrolisthesis of C3, severe kyphosis, complete cut-off in myelogram, cord compression, and myelomalacia. (A) T1-weighted image. (B) T2-weighted image. (C) T1 contrast image. (D) Myelogram. (E, F) T2-weighted axial image with corresponding sagittal cut.

A 19-year-old male presented with neck pain, spastic quadriparesis, and signs of cervical myelopathy of six months’ duration. Previously, he underwent C2-C5 laminectomy for C3 neurofibroma at an outside hospital. MRI imaging revealed severe cervical kyphosis with retrolisthesis of C3 with cord compression and myelomalacia (Figure 1).

Clinical examination revealed quadriparesis, decreased muscle bulk, grade III modified Ashworth scale spasticity in all four limbs, loss of hand dexterity, and atrophy of the thenar and hypothenar muscles. The sensations of touch, pain, and temperature were decreased from C5 downwards. In terms of pathological reflexes, Hofmann and extensor plantar were present bilaterally. The ankle and knee clonus were sustained on elicitation.

**Figure 2:**
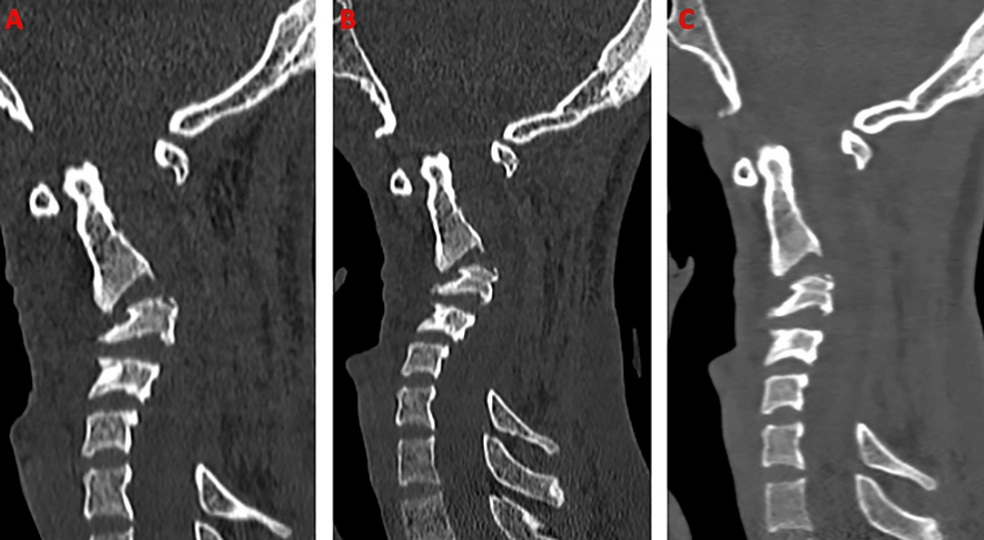
CT sagittal view showing progressive reduction of C3 retrolisthesis and cervical kyphosis with a gradual increment in the traction weights. (A) 3 kg. (B) 5 kg. (C) 8 kg.

The patient was placed on Gardener skeletal traction with an initial weight of 3 kg applied in the extension position. The weight of cervical traction was gradually increased from 3 to 8 kg over two weeks. The C3 retrolisthesis was gradually reduced with gradual straightening of the cervical curvature (Figure 2).

**Figure 3:**
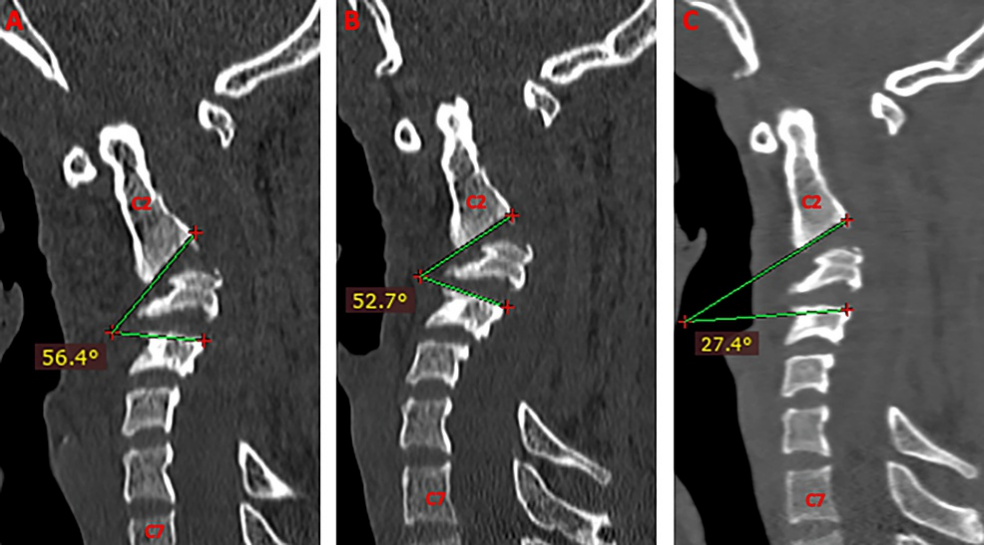
CT sagittal view showing a gradual reduction in the local kyphosis/Cobb’s angle with a gradual increment in the traction weights. (A) 3 Kg; local Cobb’s angle of 56.4 degrees. (B) 5 kg; local Cobb’s angle of 52.7 degrees. (C) 8 kg; local Cobb’s angle of 27.4 degrees.

**Figure 4:**
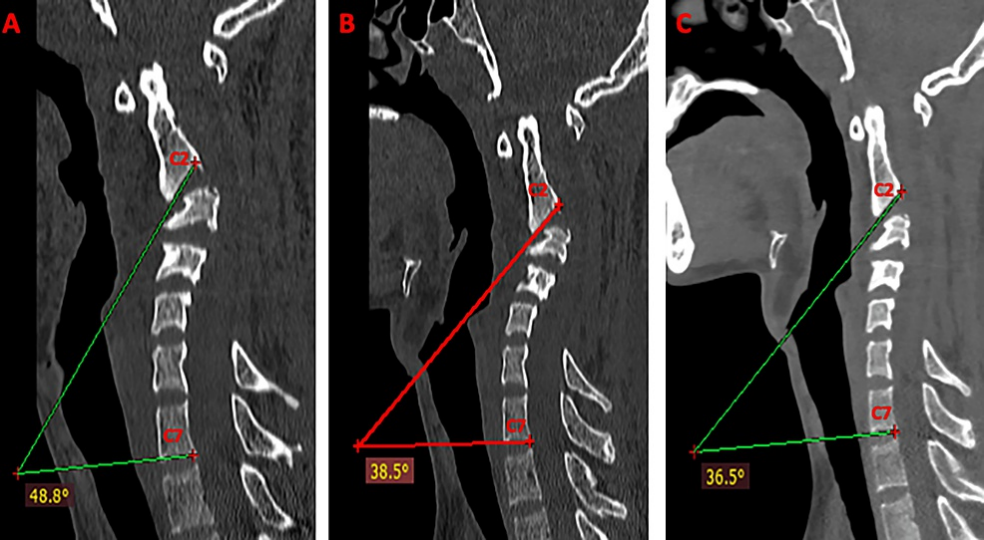
CT sagittal view showing a gradual reduction in the global kyphosis/Cobb’s angle with a gradual increment in the traction weights. (A) 3 Kg; global Cobb’s angle of 48.8 degrees. (B) 5 kg; global Cobb’s angle of 38.5 degrees. (C) 8 kg; global Cobb’s angle of 36.5 degrees.

The Cobb’s angle (local and global kyphosis angle) measurement at the point of maximum kyphosis also showed a gradual reduction in serial CTs after an increment in the weights along with improvement in the patient’s clinical condition (Figures 3, 4).

**Figure 5:**
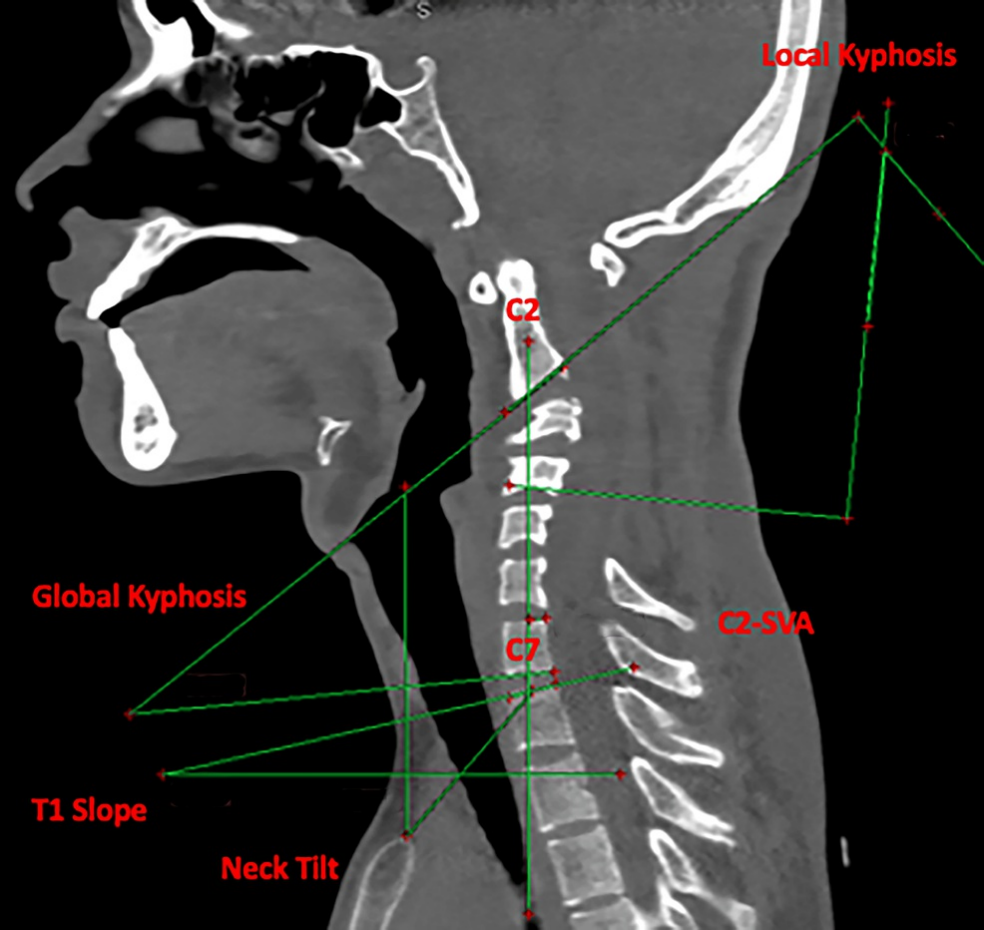
CT sagittal view showing pre-operative assessment of various cervical parameters: local kyphosis 47.4 degrees, global kyphosis 32.9 degrees, neck tilt 41.2 degrees, T1 slope 12.7 degrees, and C2-SVA 4.35 mm. C2-SVA, C2 sagittal vertical axis

The pre-operative assessment parameters included were local kyphosis angle (47.4 degrees), global kyphosis (32.9 degrees), neck tilt (41.2 degrees), T1 slope (12.7 degrees), C2 sagittal vertical axis (C2-SVA) (4.35 mm), and sigmoid type of cervical kyphosis (Figure 5).

**Figure 6:**
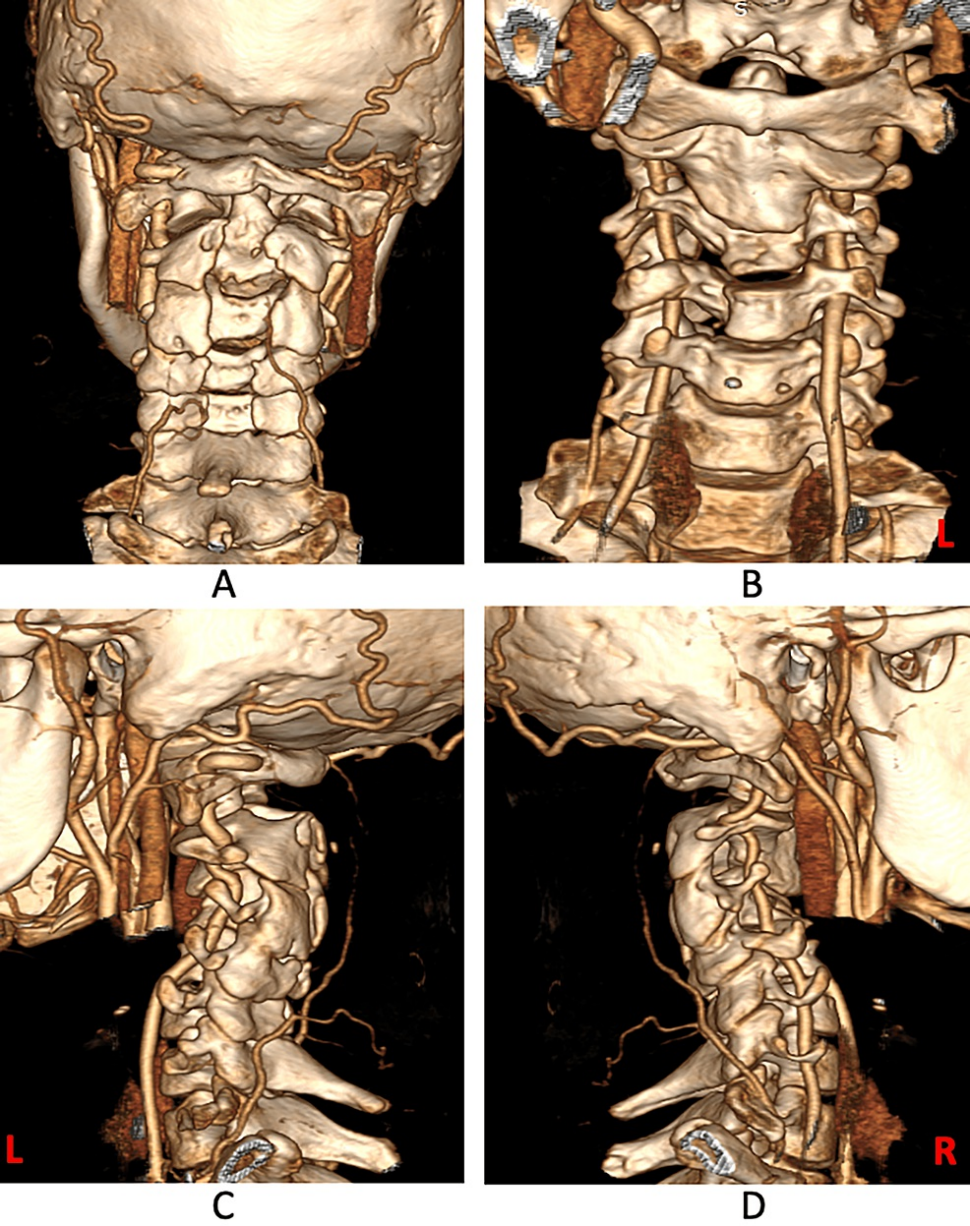
A 3D reconstruction of the CT cervical spine showing the normal caliber of the vertebral artery bilaterally. However, on the left side, the vertebral artery is seen coursing anterior to the foramen up to C5 and enters the foramen at the level of C4 vertebrae. (A) Posterior view. (B) Anterior view. (C) Left lateral view. (D) Right lateral view.

The vertebral artery was normal in caliber bilaterally. However, on the left side, the vertebral artery entered the foramen at C4 level rather than C6 (Figure 6).

**Figure 7:**
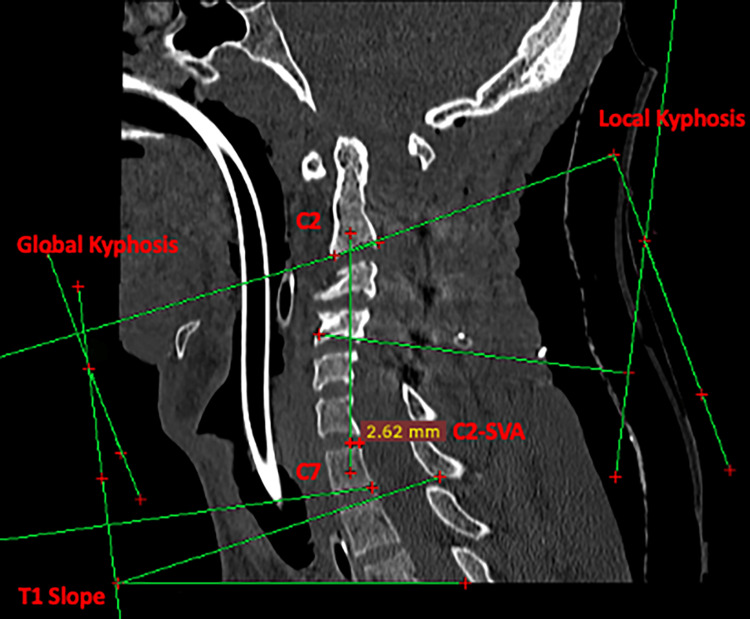
CT sagittal view showing post-operative assessment of various cervical parameters: local kyphosis 27.5 degrees, global kyphosis 14.4 degrees, neck tilt 43.6 degrees, T1 slope 18.2 degrees, and C2-SVA 2.62 mm. C2-SVA, C2 sagittal vertical axis

A posterior instrumented fusion was chosen to correct the kyphotic deformity after the gradual reduction in Cobb's angle following skeletal traction and concurrent improvement in clinical condition. The patient underwent C2 pedicle and C3-C5 lateral mass fixation in an extension position with traction weight in situ. Once all screw positions were confirmed fluoroscopically and the rod was tightened, the traction weight was removed. The follow-up scan showed a significant reduction in global and local kyphosis. The post-operative parameters were local kyphosis angle (27.5 degrees), global kyphosis (14.4 degrees), neck tilt (43.5 degrees), T1 slope (18.2 degrees), and C2-SVA (2.62 mm) (Figure 7).

The patient's modified Japanese Orthopaedic Association score improved from 10 to 16 at the six-month follow-up.

## Discussion

PLK has a complex etiology that differs in adults and children. The incidence is higher in children due to the cartilaginous nature of the anterior vertebral body, greater viscoelasticity of the ligaments, and incomplete ossification. The incidence of PLK is higher in the cervical region compared to the thoracic and lumbar regions [6]. Young age, laminectomy, pre-operative deformity, and significant facet resection are a few of the risk variables that have been linked to an increased likelihood of developing kyphosis/spinal deformity after surgery [7].

In cervical vertebrae, nearly two-thirds of the axial load passes through the posterior elements viz. facet joints, laminae, spinous process, ligaments, and musculature [8]. The PLC forms a posterior tension band, which serves as a primary spine stabilizer [9]. The posterior osseoligamentous complex consists of ligaments and the posterior vertebral arches. The articular facet joint capsule, ligamentum flavum, supraspinous ligament, and interspinous ligament make up the four main parts of the PLC. Its primary function is to restrict the spine's flexion, axial rotation, and distraction, thereby reducing the axial strain on the intervertebral discs [9,10]. In normal lordotic cervical vertebrae, the axial force (cranial center of mass) passes through the instantaneous axis of rotation (IAR) without any deviation. The PLC helps maintain the lordotic spine. However, with disc degeneration, loss of vertebral body height, or iatrogenic PLC injury, the IAR shifts ventrally with the development of the bending moment arm. A larger moment arm predisposes to the progression of the kyphotic deformity [11]. The main aspect of PLC incompetence is the rupture of the supraspinous ligament. In cases where there is inflammation in the interspinous ligament and facet disruption, the PLC is still considered competent [12]. The sequential damage to PLC involves facet joint disruption followed by interspinous ligament edema, supraspinous ligament rupture, and ligament flavum disruption [10].

The PLC rupture post-surgery increases the range of motion by 24.3%, 77.2%, and 190.7% at C2-C3, C4-C5, and C6-C7, respectively. Similarly, in the present study, the patient underwent multi-level laminectomy for C3 neurofibroma, which resulted in the development of symptomatic kyphosis with cord changes and myelomalacia. Analysis has shown the anatomical and biomechanical importance of PLC and intervertebral discs for maintaining cervical spine stability in flexion. While the disc has more of an impact on the upper cervical spine, the PLC is especially crucial for the middle and lower cervical spine's stability [13]. A cervical laminectomy by itself causes a 6%-47% incidence of PLK, which adds to late neurological deterioration rates (10%-39%). Therefore, in cases where laminectomy is anticipated, posterior cervical fusion can help prevent kyphosis [5]. Studies on the biomechanics of cervical spine post-surgery have shown that laminectomy with fusion was related to the lowest spinal cord stress and strain in flexion-extension, lateral bending, and axial rotation of the neck when compared with laminectomy and laminoplasty [14].

To circumvent the limitations of laminectomy, laminotomy, and laminoplasty were developed especially for pediatric patients. The laminoplasty significantly mitigated the likelihood of kyphosis after resection of spinal intradural tumor compared with laminectomy, although some risks still exist, attributed to the disruption of the posterior tension band, significant facet resection, or failure to reattach semispinalis cervicis to C2, which causes progressive degradation in the cervical sagittal parameters in the early post-operative period. This could lead to some distortion in health-related quality of life [15,16]. The success of laminoplasty is also dependent on favorable cervical and spinopelvic parameters. In cases with abnormal spinopelvic parameters, further disruption of PLC in laminoplasty will aggravate the spinal deformity [16]. Various modifications to the laminoplasty technique have been advocated that maintain posterior ligamentous integrity, viz. bone-to-bone ligament-preserving laminoplasty and the use of ultrasonic bone scalpel (UBS). The use of UBS creates a smaller space between the laminae and the respective lateral mass, which promotes fusion, both of which reduce the incidence of kyphosis [17]. In cases of spinal tumors (intra/extradural), we opt for laminotomy and tumor excision. However, when a large window needs to be created, we opt for modified en bloc laminoplasty using a high-speed drill bone cutter (F1) to reduce bone loss along with preservation of PLC or laminectomy with posterior cervical fusion to prevent the development of late kyphosis.

The imaging for cervical spinal deformity includes a cervical X-ray (anteroposterior and lateral views), a full 36-inch X-ray, a CT scan, and an MRI. In addition, surgical planning involves a detailed assessment of the cervical and spinopelvic parameters. Pre-operative planning should incorporate the neurological status, bone condition, deformity characteristics, ankylosis, previous surgeries, presence of degenerative changes at the proximal or distal vertebral level, course of the vertebral artery, and the presence of any medical comorbidities [18]. The pre-operative imaging parameters should be considered while deciding the degree of correction for the spinal deformity. Additionally, every effort should be made to restore the sagittal balance as close to neutral as possible. The literature evidence suggests that T1 slope - cervical lordosis < 15 degrees, C2-SVA < 40 mm, and chin brow vertical angle (CBVA) between -10° and +20° are generally acceptable [19].

The purpose of axial traction is to safely and effectively treat severe spinal deformities. Correcting severe scoliosis too quickly can increase the risk of neurological damage. Gradually increasing traction over several weeks can partially correct kyphotic deformities. Surgeons have varying opinions on the application of traction for deformity correction. However, using pre-operative skeletal traction allows for gradual correction before the final surgery. Research by Park et al. aimed to determine the optimal duration of traction to maximize pre-operative correction and reduce halo-related issues. The majority of sagittal corrections occurred within two weeks of applying traction, and no permanent neurological complications were reported [20]. The same period of skeletal traction was applied in the present study and a reduction of 18.5 degrees was noted in the Cobb’s angle. However, traction-related complications should always be known, viz. ileus, respiratory complications, pin loosening, pin site infection, transient brachial plexus injury, hypoglossal nerve damage, poor patient tolerance, prolonged immobility, and bed sores.

**Figure 8:**
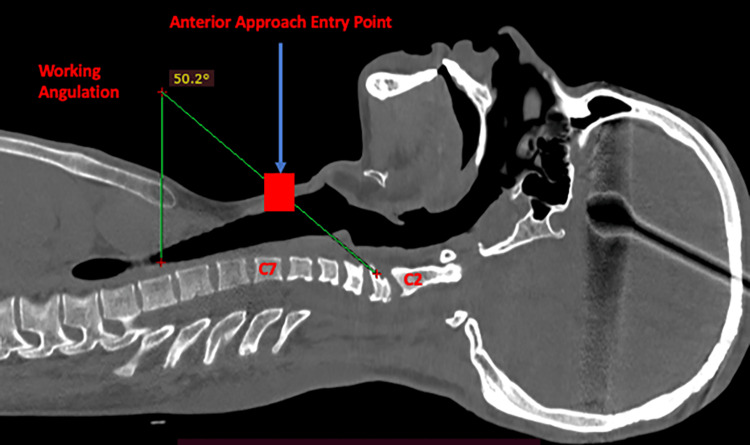
CT sagittal view showing difficulties encountered during an anterior approach with a working angulation of 50.2 degrees.

For correcting PLK, either an anterior, posterior, or combined approach may be used. However, in this case, the anterior approach was not chosen due to the need for high cervical corpectomy, which was limited by the overlying mandible bone and an acute operative angle that would restrict visualization of the posterior vertebral body (Figure 8).

The goal of this surgery was to correct the kyphotic deformity to bring Cobb’s angle to as close to neutral as possible and decompress the spinal cord and the nerve roots due to severe compression. Any overcorrection would mandate the fixation of C1 and the occiput also. A posterior cervical fusion was performed owing to severe kyphosis, which we believe should have been carried out at the time of first surgery during multilevel laminectomy to avoid late development of symptomatic kyphosis. The posterior approach was carried in line with the cervical deformity management algorithm proposed by Steinmetz et al. (anterior compression > 3 levels, flexible deformity showing reduction on skeletal traction) [21]. As a result, there was a decrease in both the local and global kyphosis angles, C2-7 SVA, NT/T1 slope ratio, and CBVA, while the T1 slope increased, indicating the return of the cervical lordosis. The considerable changes in the cervical parameters were also evident clinically, as there was a reduction in spasticity and an improvement in weakness.

## Conclusions

The present case highlights the importance of preserving the PLC as a major spine stabilizer and preventing PLK. Surgical interventions such as laminoplasty, laminotomy, and laminectomy with posterior cervical fusion aim to mitigate the risk of kyphosis. Patients undergoing multilevel laminectomy or significant facet resection should be supplemented with spinal fusion to prevent the development of late kyphosis. For severe kyphotic deformity, the application of skeletal traction can be considered. However, the occurrence of potential complications should be known. In addition, this report highlights the role of thorough pre-operative planning, assessment of cervical and spinopelvic parameters, operative approaches, and technical nuances required to restore the cervical sagittal balance.
